# Supplementary figures and images for: The Japanese encephalitis virus NS1′ protein facilitates virus infection in mosquitoes
Source: PLoS Negl Trop Dis. 2025 Jan 27;19(1):e0012823. doi: 10.1371/journal.pntd.0012823 (PMC11781682; doi:10.1371/journal.pntd.0012823)

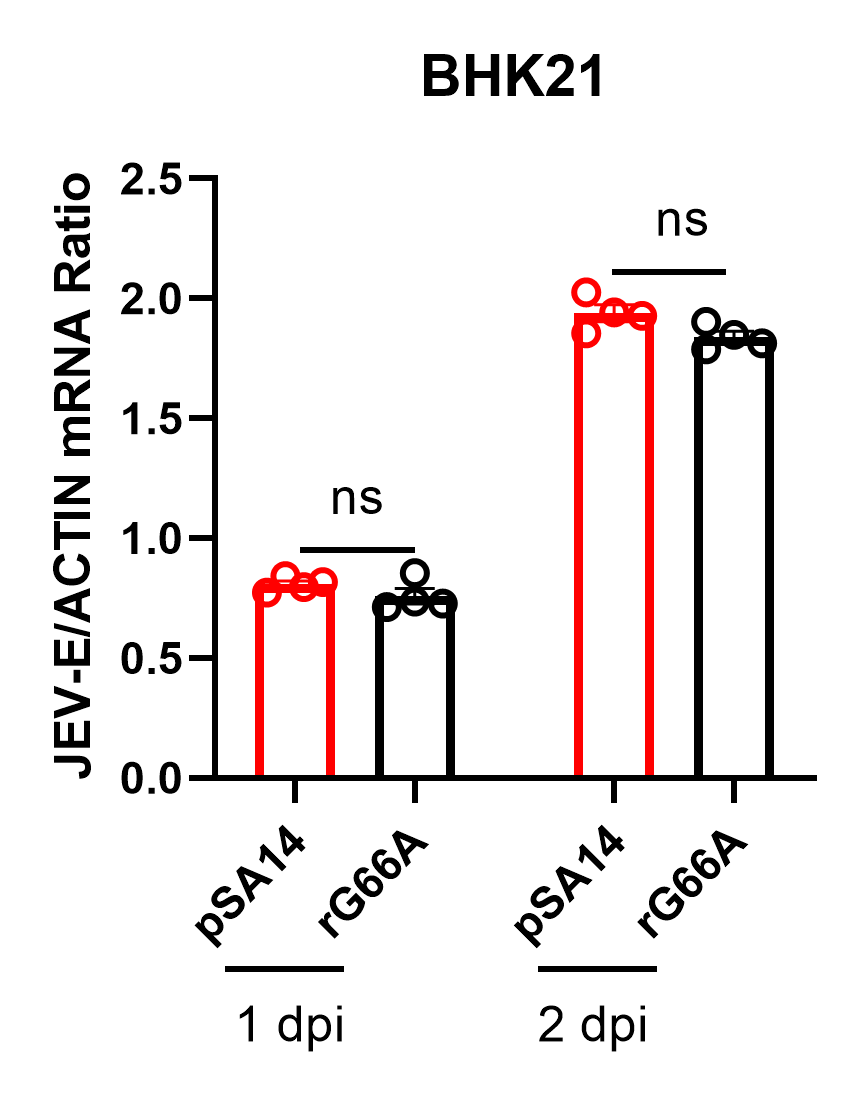

Supplement: S1 Fig — BHK21 cells were infected with pSA14 or rG66A at an MOI of 0.1. The cell lysates were harvested at 1day or 2days after infection. The mRNA levels of E genes were determined by qPCR. The data are presented as the mean ± SEM. A nonparametric Mann-Whitney test was used for the statistical analyses. *P < 0.05, **P < 0.01. The experiments were biologically repeated at least 3 times with similar results. (TIF) [file pntd.0012823.s001.tif]

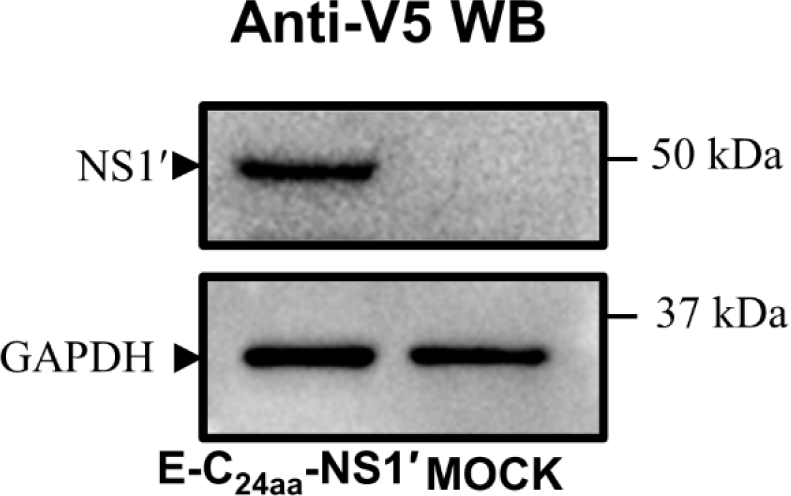

Supplement: S2 Fig — The NS1′ genes were cloned into a pAC-V5-HisA vector. C6/36 cells were transfected with empty vectors or vectors expressing NS1′. Expression of empty vector and vectors expressing NS1′ were analyzed by western blotting using anti-V5. (TIF) [file pntd.0012823.s002.tif]

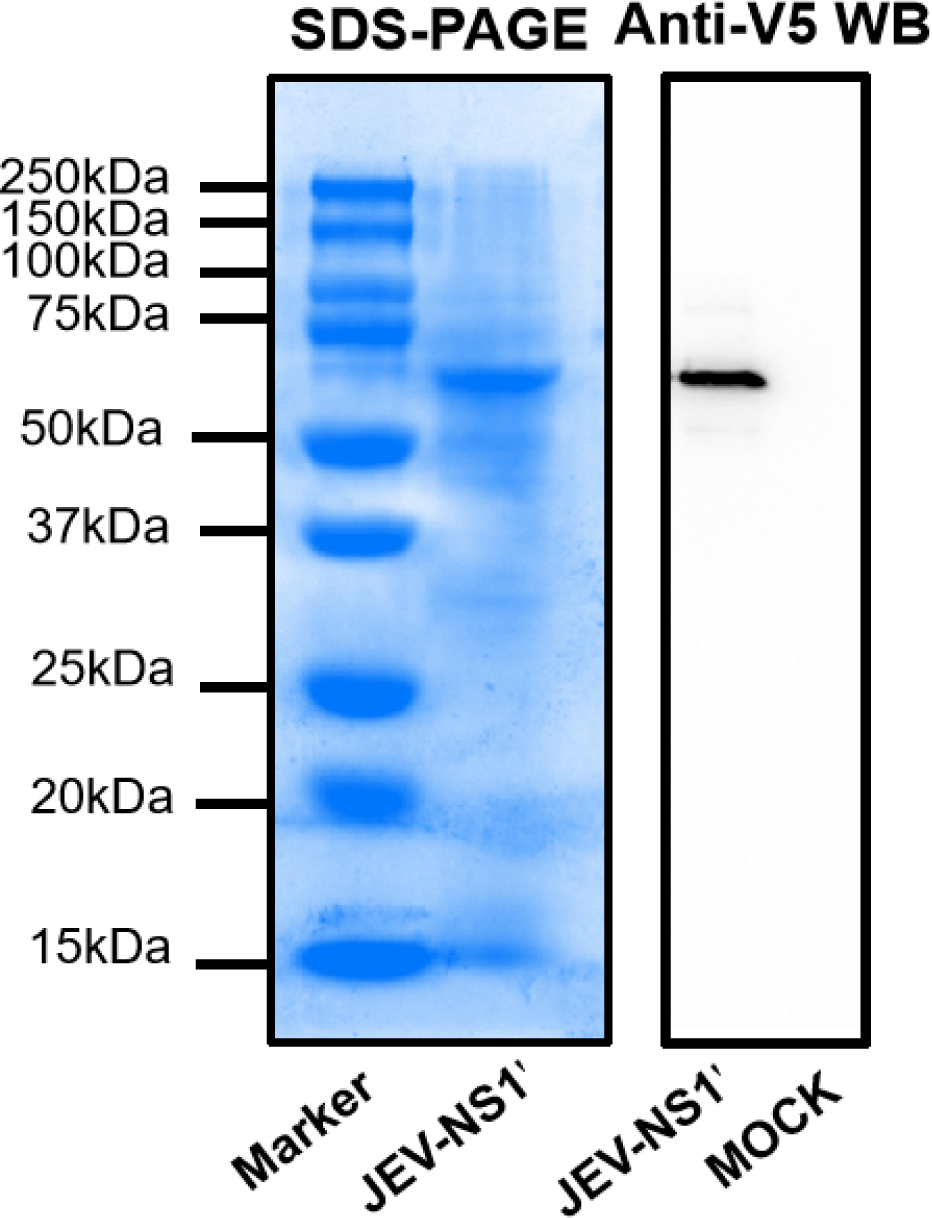

Supplement: S3 Fig — The NS1′ genes were cloned into pMT/BiP/V5-HisA vector. And NS1′ protein was expressed and purified on Econo Fit Nuvia IMAC column (Left panel). Protein expression was evaluated using an anti-V5-HRP mAb (Right panel). (TIF) [file pntd.0012823.s003.tif]

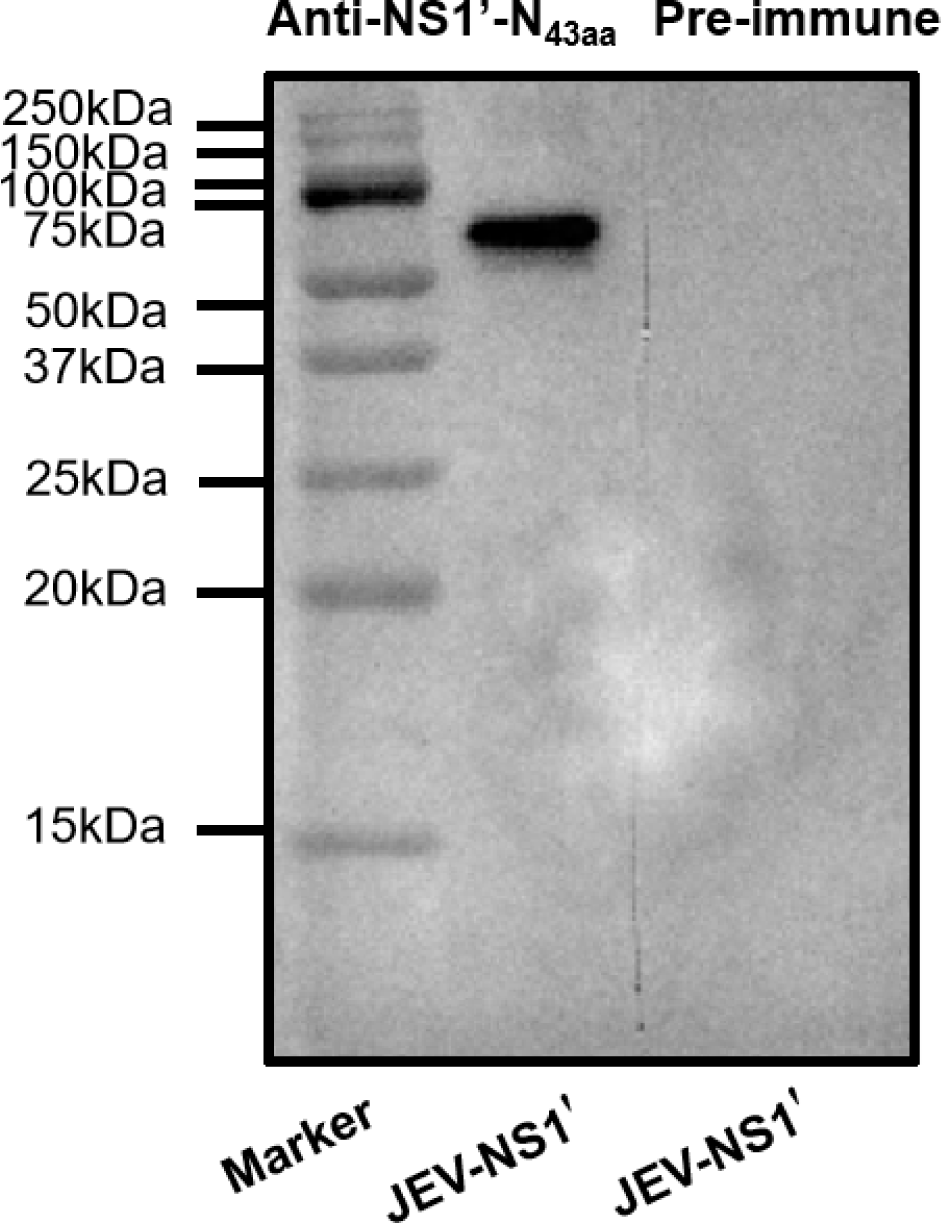

Supplement: S4 Fig — The last 43 amnio acid of NS1′ (ΔNS1′43aa) were synthesized in Hefei SynthBiological Engineering Company. The mice were immunized with 8ug ΔNS1′43aa each. And the The mice serum was stored at −80 °C. The JEV infected cells lysates weas loaded into each lane. The same samples probed by mouse pre-immune antibody served as a negative control. (TIF) [file pntd.0012823.s004.tif]

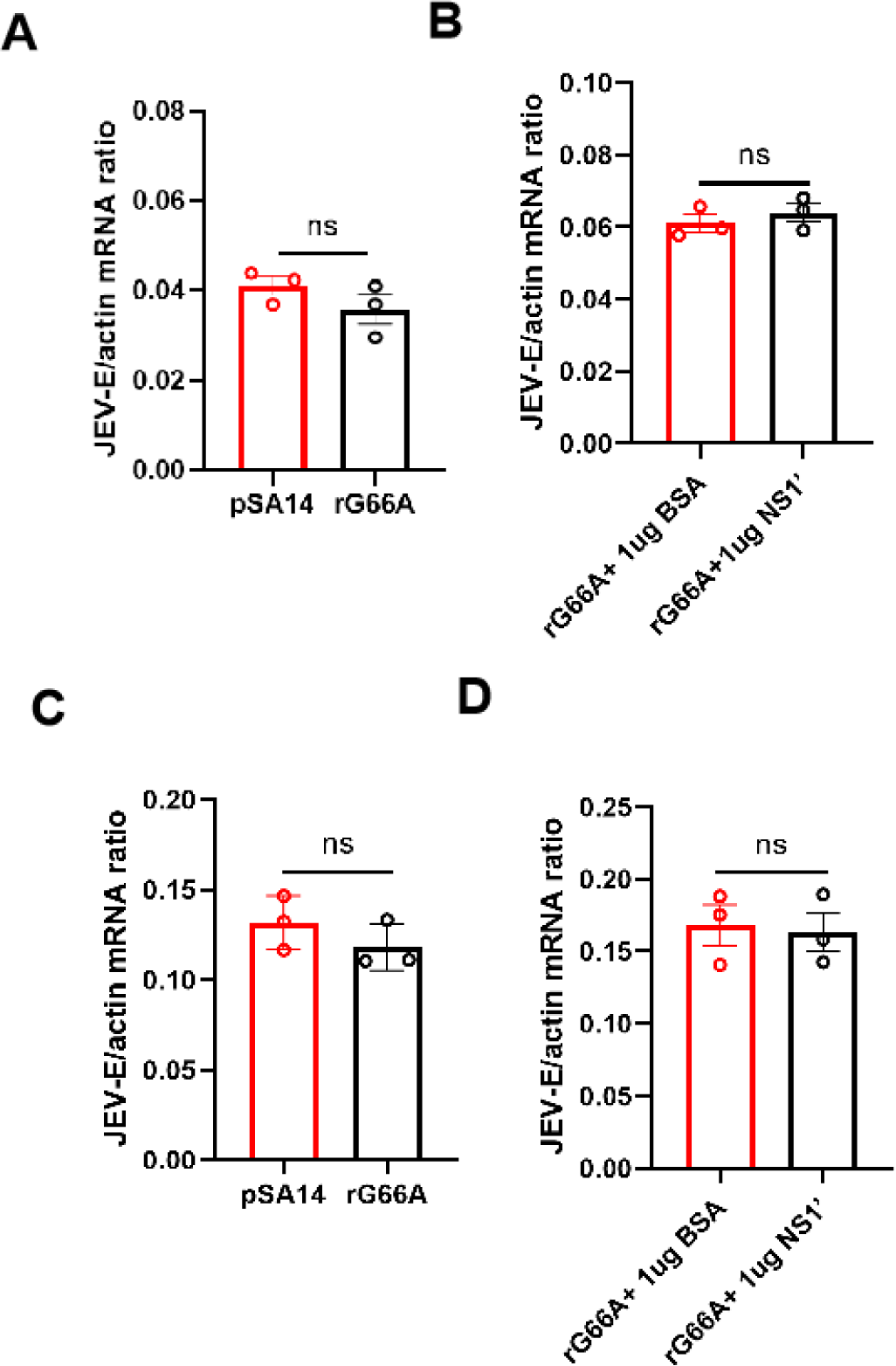

Supplement: S5 Fig — (A and B) Viral attachment was assessed by RT-qPCR. (A) C6/36 cells were incubated with JEV at an MOI of 5 for 1 h at 4 °C. The unbound virus was removed by washing with PBS. After washing with PBS, the cells were assessed by RT-qPCR. (B) C6/36 cells were incubated with rG66A viron and 1µg NS1’ protein at an MOI of 5 for 1 h at 4 °C. (C and D) Viral internalization into cells was assessed by RT-qPCR. (C) For the internalization assay, after JEV adsorption at 4 °C for 1 h, the cells were washed with PBS and subsequently incubated to 30 °C for 1 h to allow virion internalization. After washing with PBS, the cells were treated with proteinase K to remove noninternalized virions. (D) C6/36 cells were infected with rG66A virus and 1 µg NS1’ protein at an MOI of 5. (A–D) The data are presented as the mean ± SEM. The nonparametric Mann-Whitney test was used for statistical analysis. **P < 0.01; n.s., not significant. (TIF) [file pntd.0012823.s005.tif]

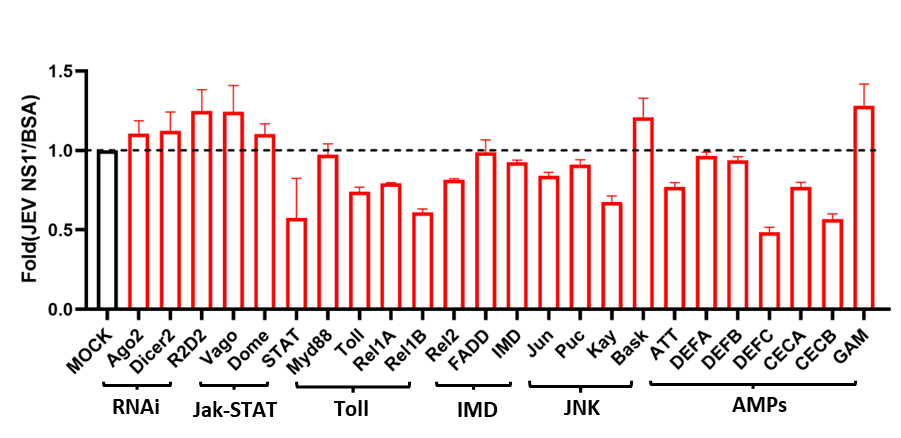

Supplement: S6 Fig — The expression levels of the immune-related genes were assessed by qPCR at 48 hours post micro-injection. Gene regulation is represented as the mRNA ration between NS1′ injected and BSA injected in rG66A infected mosquitoes. The primers used are listed in S1 Table. The data are presented as the mean ± SEM. The nonparametric Mann-Whitney test was used for statistical analysis. **P < 0.01; n.s., not significant. (TIF) [file pntd.0012823.s006.tif]

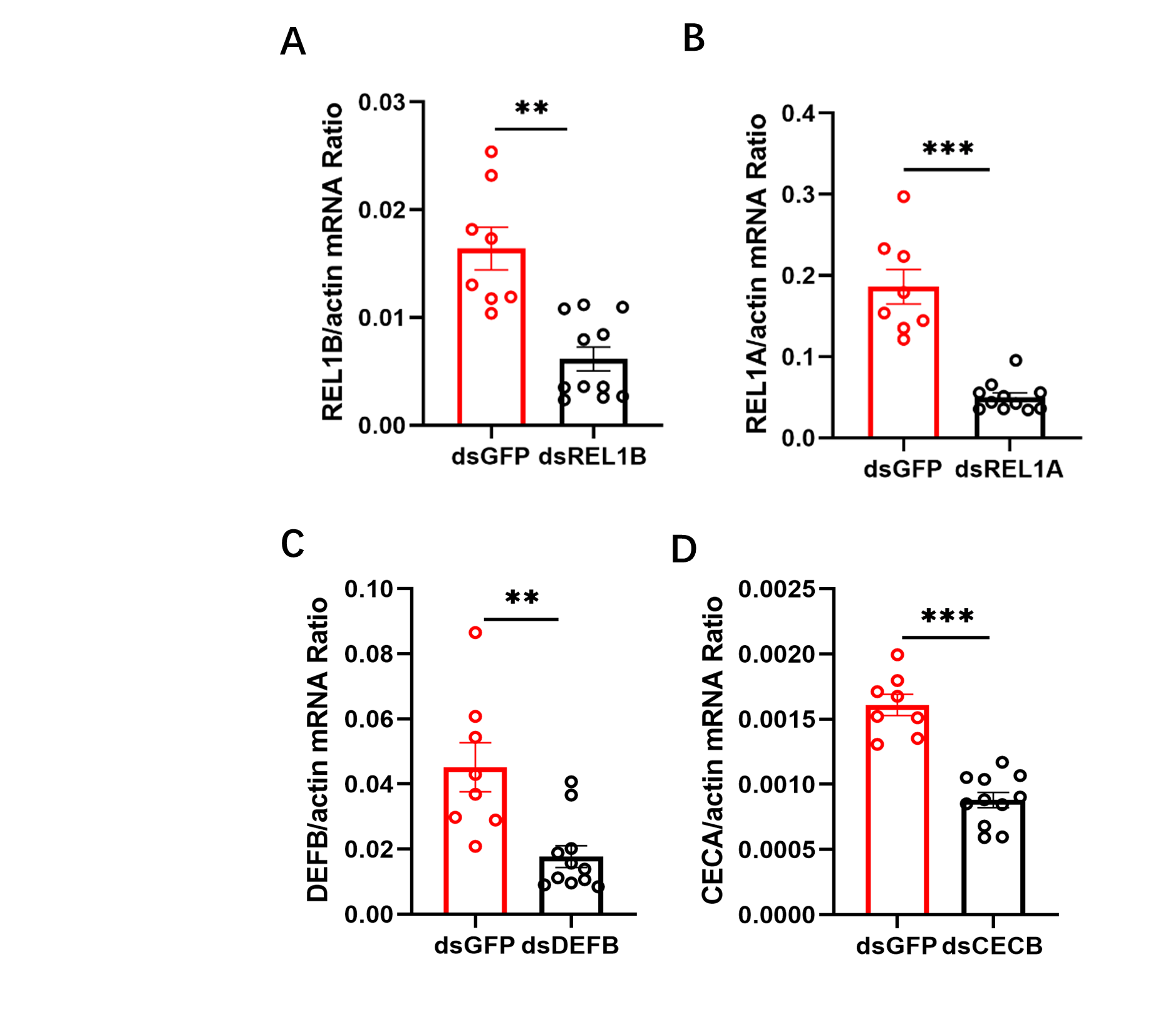

Supplement: S7 Fig — (A–D) Total RNA was extracted, gene levels were detected by RT-qPCR. Gene expression was normalized to the Culex quinquefasciatus actin gene. Data are represented as mean ± SEM. in each group and analyzed using the nonparametric Mann Whitney test. **P < 0.01, ****P < 0.0001. (TIF) [file pntd.0012823.s007.tif]

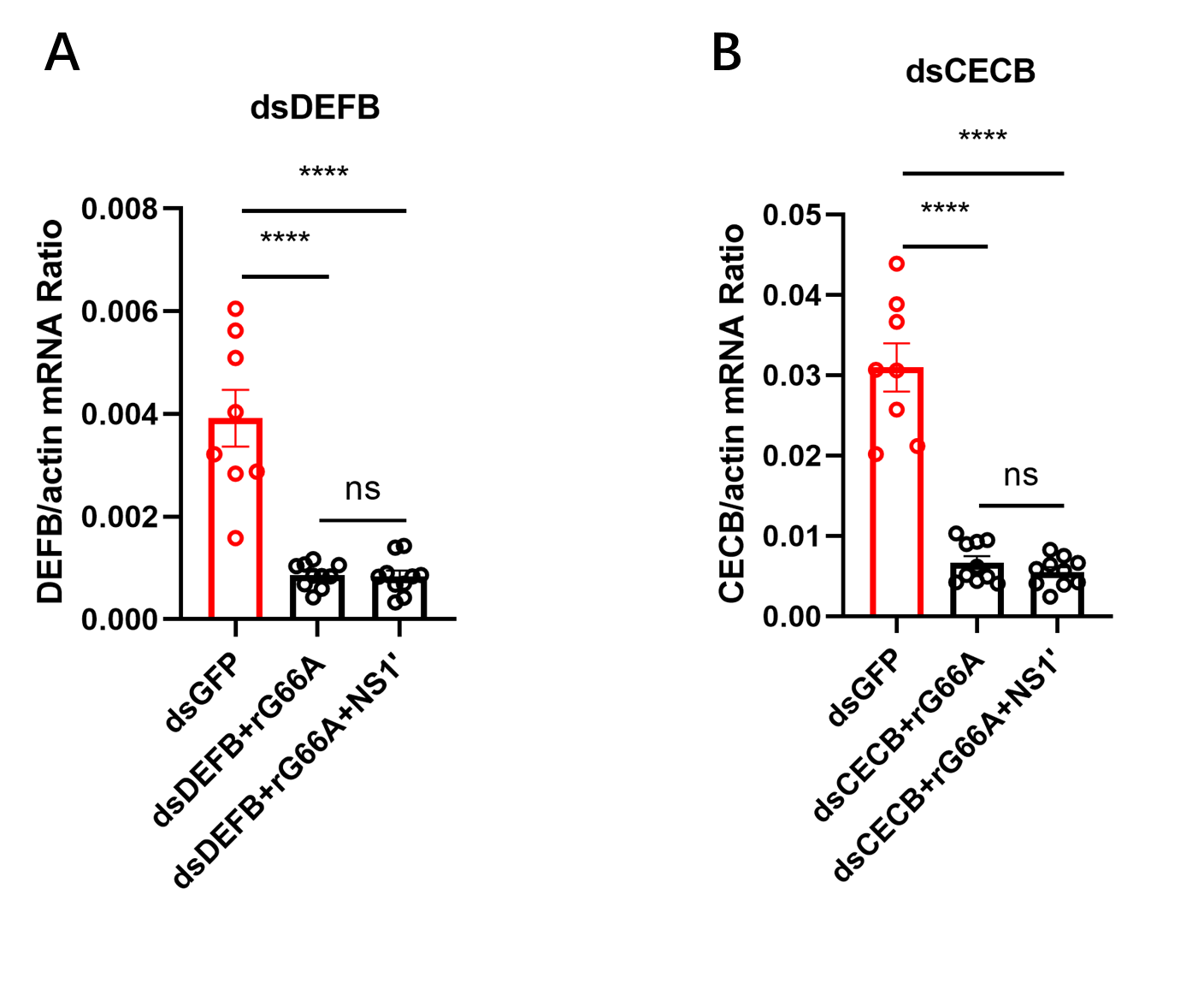

Supplement: S8 Fig — (A and B) Total RNA was extracted, gene levels were detected by RT-qPCR. Gene expression was normalized to the Culex quinquefasciatus actin gene. Data are represented as mean ± SEM. in each group and analyzed using the nonparametric Mann Whitney test. **P < 0.01, ****P < 0.0001. (TIF) [file pntd.0012823.s008.tif]
