# Supplementary material for: The Japanese encephalitis virus NS1′ protein facilitates virus infection in mosquitoes
Source: PLoS Negl Trop Dis. 2025 Jan 27;19(1):e0012823. doi: 10.1371/journal.pntd.0012823 (PMC11781682; doi:10.1371/journal.pntd.0012823)
Supplement: Attachment — Submitted filename: response to referees 12.8.docx [file pntd.0012823.s011.docx]

*Dear Editors,*

*Thank you for the comments on our manuscript (PNTD-D-24-01139) titled "* *The Japanese encephalitis virus NS1′ protein facilitates virus infection in mosquitoes". We have taken all of the comments into consideration and have revised our manuscript accordingly. We believe that our additional experiments and modifications of the text have substantially improved the manuscript.*

*We were encouraged by the reviewers’ positive comments, such as "* *This study is interesting because it provides new insights into JEV transmission mechanisms through the viral non-structural protein.", and" Conclusions authors claimed in this study are interesting and contribute in this research field." (Reviewer #1), "* *In this study, the authors explore the potential role of the NS1 protein (or more precisely NS1') in mosquito infection, by leveraging on NS1'-defective virus (rG66A). While the results are interesting " (Reviewer #3). The reviewers also provided important suggestions to improve the manuscript. Along with this letter, we provide point-by-point responses to the queries raised by the editor and reviewers.*

**Responses to** **Reviewer #1:**

**#1-1.** *Although I agree that the NS1′ protein promotes virus infection and replication in mosquitoes, my main concern is that the figures and results presented here do not fully corroborate the authors' findings, particularly how NS1′ regulates the JEV life cycle (attachment, entry, RNA replication, assembly, or release). I hope addressing the following comments will help improve the manuscript.*

**#1-1 Answer:** Thank you for the reviewer's comments. Based on the reviewer’s valuable guidance, we have supplemented those experiments including the regulation of JEV attachment, entry, and RNA replication by NS1’. As shown the result below, NS1 protein does not affect the adsorption and internalization processes of JEV but instead influences the early stage of viral replication. We have added those results in Figure 2I-J, and Supplement Fig. 5.

**#1-2.** *Line 232 needs references or evidence.*

**#1-2 Answer:** Thank you for your suggestion. We have added the reference in the manuscript (Line 246) and the references list.

**#1-3.** *Figure 1B–E needs quantification data.*

**#1-3 Answer:** Thanks for your meaningful comment. We apologize for the lack of quantification results in our previous manuscript. To address the reviewer’s concern, we included quantification results in the revised document in Figure 1B-E.

**#1-4.** *In Figure 2, the authors performed only qPCR. The entire phenotype of NS1′ protein for viral replication is not very apparent, since all the data were significant but not so high (about 2–3 fold change in RNA levels). How NS1′ regulates JEV propagation in mosquito cell lines remains unclear. The ratios of viral attachment on cell membrane and viral titers should be quantified.*

**#1-4 Answer:** Thank you for the reviewer’s comments. According to the reviewer’s comments, we examined the effect of the NS1’ protein on the viral life cycle. As shown in the result below, NS1’ protein does not affect the adsorption and internalization processes of JEV but instead influences the early stage of viral replication. We have added those results in Figure 2I-J, and Supplement Fig. 5. Besides, we added the viral titer by plaque assay that was incorporated in Figure 2B and 2D. These results are consistent with the results obtained by qPCR.

**#1-5.** *Authors added NS1′ protein into virus-infected cells. Authors should confirm NS1′ was found intracellular.*

**#1-5 Answer:** Considering the Reviewer’s suggestion, intracellular levels of NS1’ protein were measured using western blotting after the protein was added to the supernatant. As shown in the results below, the NS1’ protein was detected inside the cells, indicating that it can be transported into cells through a specific mechanism (Figure R1). previous studies have shown that NS1 protein forms a lipoprotein particle and can bind to mosquito cells and be internalized via a scavenger receptor B1-like factor (PMID:34986002 and PMID:21518917). Consistent with NS1, NS1’ protein has three forms, a monomer, dimer (membrane-bound protein), and hexamer (secreted protein) (PMID: 25614585 and PMID: 25392222). Therefore, we speculate that the NS1’ protein may enter cells through the same mechanism as the NS1 protein.

**#1-6.** *In Line 386, explain how NS1′ suppresses AMPs via the Toll pathway? If the AMP pathway is affected by NS1′, how did AMP impair viral RNA as shown in Figure 2?*

**#1-6 Answer:** Thank you for the reviewer's comments. In mosquitoes, the Toll and IMD pathways are involved in the defense against various arthropod-borne viruses infections (PMID: 16203210; PMID:18604274; PMID: 20079370; PMID: 23861987). Unlike the NS1 protein, which inhibits ROS and the JAK-STAT pathway (PMID: 27562253), the NS1’ protein suppresses the Toll and IMD pathways components, thereby inhibiting AMPs expression. The mechanism by which the NS1’ protein inhibits AMPs *via* the Toll pathway warrants further investigation. The NS1’ protein is capable of extracellular secretion and may interact with extracellular proteins associated with the Toll pathway, including leucine-rich repeat-containing proteins, clip-domain serine proteases (PMID: 26688791). Moreover, the NS1’ protein is able to be internalized into mosquito cells, potentially influencing the regulation of downstream genes within the Toll pathway. nonetheless, additional research is needed to elucidate the specific regulatory roles of NS1’ on these genes and to identify the proteins with which it interacts.

Most insect AMPs are small with activities against bacteria or fungi, while some AMPs also show activities against viruses (PMID: 24811407). A majority of AMPs exhibit antiviral activity primarily by inhibiting viral attachment and membrane fusion, destroying the viral envelope, or suppressing viral replication (PMID: 31375212). Melittin and cecropin, two well-characterized AMPs, exhibit antiviral activity by inhibiting the expression of HIV-related genes (PMID: 9568968). ATT and dipterich have been shown to exert an antiviral activity by controlling viral RNA synthesis during SINV infection in *Drosophila* (PMID: 23365449). Multiple AMPs could be upregulated in mosquitoes following flavivirus infection, such as DEFA, DEFE, and CECE (PMID: 29109710). In *Aedes aegypti* AaSR-C/AaMCR induces AMPs expression to oppose DENV infection (PMID: 24722701). We have added this discussion of this content in the revised manuscript (Line 521-542).

**#1-7.** *In Figure 4, the authors showed that NS1′ administration downregulated the mRNA expression levels of genes associated with the Toll pathway and several antimicrobial peptides (AMPs) in mosquitoes, using the RNA-seq and qPCR. However, the effect of these AMPs on JEV replication is unclear. The author should measure the expression levels of AMPs and genes associated with the Toll pathway in pSA14- or rG66A-infected cells and mosquitoes. Moreover, NS1′ protein administration cannot ameliorate the effect of NS1 region, because previous report showed that NS1′ shares a similar cellular distribution with NS1(19). Since the authors demonstrated that the peptides of ΔNS1′44aa enhanced viral replication in mosquito cells (Figure 2E), I recommend the use of ΔNS1′44aa for microinjection in Figure 4.*

**#1-7 Answer:** We recognize the reviewer's concerns. We measured the expression level of some AMPs and the toll pathway components after pSA14 or rG66A infected in mosquitoes and C6/36 cell line. As shown in the result below, following infection with the pSA14 virus, the expression of Toll pathway-related genes and AMPs in mosquitoes was significantly lower than in the rG66A virus group, indicating that the WT virus can indeed suppress this pathway (Figure R2 and Figure R3). We administered the 43aa peptide to mosquitoes to investigate potential alterations in the Toll pathway and AMP expression. The findings indicated that the peptide was capable of partially suppressing the Toll pathway and AMP expression; however, its inhibitory effect was less pronounced than that observed with the complete NS1' protein (Figure R4). This may be due to the lower stability of the peptide compared to the full protein.

**#1-8.** *Why did the author use introduced ΔNS1′44aa but not ΔNS1′52aa (the last 52 unique amino acids of NS1′ protein) as reported previously (PMID: 31996459)?*

**#1-8 Answer:** Thank you for the reviewer’s comments and we are sorry for not expressing ourselves clearly and the mistake. The NS1’ protein is an additional form of NS1 protein with 52aa extension, which including the N-terminal 9aa of NS2A and 43aa after the frameshift (PMID: 19906906; PMID: 22504173; PMID: 23760245). Therefore, the 43aa are unique to NS1’. In fact, we synthesized a peptide fragment consisting of the C-terminal 43aa of the NS1’protein. Subsequently we generated an antibody specifically targeting the last 43aa to selectively block its function. We have corrected this error in the revised manuscript.

**#1-9.** *Previous report (PMID:* *24443559) showed that NS1′ protein efficiently co-localizes with NS5 protein and increases viral RNA levels in avian cells. Co-immunoprecipitation assays of NS1′ and NS5 in BHK21 and mosquitoes cell line are needed.*

**#1-9 Answer:** Thank you for the reviewer's suggestion. Since the interaction between NS1’ and NS5 in BHK21 cells has already been reported (PMID: 24443559), we did not conduct further experiments to investigate it. We used co-immunoprecipitation (CO-IP) in mosquito cells to examine whether NS1’ and NS5 colocalize following viral infection. As shown the result below, NS1’ protein colocalized with NS5 protein (Figure R5). Similar with in avian cells, the interaction of NS1’ and NS5 may influence effective viral RNA replication in mosquito cells.

**#1-10.** *All data lacks how P values were determined by Prism software.*

**#1-10 Answer:** Thank you for the reviewer's suggestion. P values were determined using Mann-Whitney testing. We have revised them in the revised manuscript (Line 236-237, 351-353, 408-410, 464-466).

**Responses to Reviewer #2:**

**#2-1.** *The authors evaluated the function of NS1’ in JEV infection in mosquitoes. The hypothesis was based on NS1’ pro-viral function in mosquitoes previously demonstrated for WNV. The study starts with the production of a NS1’ deficient JEV infectious clone. The authors then show that NS1’-deficient virus replicates less in mosquito cells and that the reduced infection is compensated by NS1’ complementation through either plasmid expression or protein supplementation. The last 44 aa of NS1’ are sufficient to reproduce the full-length NS1’ enhancing effect and the pro-viral effect is inhibited by anti-NS1’ antibody. They support NS1’ pro-viral effect in vivo by quantifying an increased infection in mosquitoes after infection by injection and oral feedings. Using complementation and immuno inhibition approaches as in cells, they confirm that NS1’ is responsible for the infection enhancement. Finally, they conducted RNA seq in NS1’ injected-mosquitoes and found downregulation of AMPs and Toll, suggesting a mechanism. Overall, the study strongly demonstrates that NS1’ enhances infection in mosquitoes, although the discovery of NS1’ function in mosquitoes was previously discovered for WNV, limiting the novelty. The suggestion about the associated mechanism are interesting less well supported and would require further discussion. Specifically, the authors should discuss whether the downregulation of AMPs and Toll components can be caused by the NS1 sequence that is conserved in NS1’*.

**#2-1 Answer:** Thank you for the reviewer’s comments. The mechanisms were thoroughly discussed in the discussion section (line 546-561). In the experiment, we introduced the 43aa peptide into cells and found that it enhanced viral replication. Furthermore, blocking its function with an antibody targeting the 43aa sequence inhibited viral proliferation. This indicates that the regulation of the Toll pathway is not caused by the conserved sequence in NS1. We have added discussion of these concerns mentioned by the reviewer to the Discussion section (line 486-493).

**#2-2.** *Short title is not clearly written.*

**#2-2 Answer:** Thank you for the reviewer’s comments. We have revised the title in the revised manuscript (line 243, 273, 355, 412).

**#2-3.** *Fig. 1: It should be noted that there remains a band for NS1’, although I agree it is clearly reduced.*

**#2-3 Answer:** Thank you for the reviewer’s comments. To address reviewer’s concern, we included quantification results in the revised document in Figure 1B-E.

**#2-4.** *Fig.4. The color code is confusing. Generally, green means it is increased. Please reverse the color code. Add the gene names in the heatmap.*

**#2-4 Answer:** Thank you for pointing out this problem in our manuscript. The RNA sequencing data have been presented by volcano plot in the Figure 4A-B and the gene names have been added in the map. Additionally, we modified the color scheme of the heatmap, with blue indicating downregulation (Figure 4C).

**#2-5.** *l. 48: a critical*

**#2-5 Answer:** Thank you for the reviewer’s comments. We have corrected that in the revised manuscript (Line 48).

**#2-6.** *l. 57: the sentence is not clear. Correct “and” for “which”*

**#2-6 Answer:** Thank you for the reviewer’s comments. We have made the change in the revised manuscript (Line 57).

**#2-7.** l. 62: “child as the primary host” the formulation is suprising. Children are not a primary host but maybe the most affected by the infection.

**#2-7 Answer:** Thank you for the reviewer’s comments. We have made the correction in the revised manuscript (Line 62).

**#2-8.** *l. 75: replace solitary with unique.*

**#2-8 Answer:** Thank you for the reviewer’s comments. We have made the change in the revised manuscript (Line 75).

**#2-9.** *l. 99: remove “in”*

**#2-9 Answer:** Thank you for the reviewer’s comments. We have made the change in the revised manuscript (Line 99).

**#2-10.** *l. 111: add reference for the antiviral activity of AMPs*

**#2-10 Answer:** Thank you for the reviewer’s comments. We have added reference to the revised manuscript (Line 111).

**#2-11.** *l. 132. Add the reference for the cell line.*

**#2-11 Answer:** Thank you for the reviewer’s comments. We have added reference to the revised manuscript (Line 133).

**#2-12.** *l. 139-140: if not providing a brief description of the methods, refer to references.*

**#2-12 Answer:** Thank you for the reviewer’s comments. We have added reference to the revised manuscript (Line 140).

**#2-13.** *l. 151: minon?*

**#2-13 Answer:** Thank you for pointing out this problem in our manuscript. We are sorry for our incorrect writing and it is rectified as “30 min on” in the revised manuscript (Line 151).

**#2-14.** *l. 161: subtitle is badly written*

**#2-14 Answer:** We thank the reviewer for pointing out this issue. The subtitle has been replaced by “JEV NS1′ protein generation in a *Drosophila* expression system” (Line 161).

**#2-15.** *l. 173-179: centrifugation at 25 000 g should not precipitate virions… did the authors make an error in centrifugation speed?*

**#2-15 Answer:** Thank you for the reviewer’s comments. Because viruses are macromolecular structures, they can be concentrated by a relatively short centrifugation step. DENV and retroviral particles were able to be purified by centrifugation at 25,000g and 4 °C for 6h (PMID: 27562253; PMID: 18265280). Therefore, we can obtain JEV virions using this method. We have added references to the revised manuscript (Line 175).

**#2-16.** *l. 189: provide the primer sequences*

**#2-16 Answer:** Thank you for the reviewer's comments. The primers and probes used are shown in Supplementary Table 1.

**#2-17.** *l. 230-245: this whole section should be thoroughly improved for English.*

**#2-17 Answer:** Thank you for the reviewer's comments. We have comprehensively revised this section by using an English language editing service (Line 245-259).

**#2-18.** *l. 266: replace “couldn’t” with “did not”*

**#2-18 Answer:** Thank you for the reviewer's comments. We have replaced *“couldn’t” with “did not”* in the revised manuscript (Line 282).

**#2-19.** *l. 286: precise what hypothesis is supported here*

**#2-19 Answer:** We agree with the reviewer's suggestion. We have clearly articulated our hypotheses at the end of this paragraph in the revised manuscript (Line 301-302).

**#2-20.** *l. 330: not clear how mosquitoes were infected here: injection or oral feeding? Please precise.*

**#2-20 Answer:** Thank you for the reviewer's comments. The mosquitoes were infected by thoracic microinjection in this experiment. We emphasized the route of infection in line 374 .

**#2-21.** *l. 373: mosquitoes were dissected but what tissues were analysed? Also, precise*

**#2-21 Answer:** Thank you for pointing out this problem in our manuscript. We are sorry for our mistake. We used the whole mosquitoes for RNA-seq analysis and we replaced “dissected” with “sacrificed” in the revised manuscript (Line 417).

**#2-22.** *l. 377: JNK is also an anti-viral pathway. Add the regulation of the components of this pathway: c-Jun, Kay, Puckered, basket.*

**#2-22 Answer:** Thanks for the reviewer's suggestion. In the RNA-seq analysis, genes associated with the JNK pathway exhibited no significant changes. As a result, the genes from this pathway were not included in the heatmap. At the same time, we added the regulation of JNK pathway components in Figure 4D. The results indicated that genes associated with the JNK pathway are not regulated.

**#2-23.** *l. 416: there is one to many “serological”*

**#2-23 Answer:** Thank you for pointing out the mistake in our manuscript. We have deleted the superfluous word in the revised manuscript (Line 495).

**#2-24.** *l. 432: intrathoracic*

**#2-24 Answer:** Thank you for pointing out the mistake in our manuscript. We have corrected it in the revised manuscript (Line 511).

**#2-25.** *l. 442: references are missing*

**#2-25 Answer:** Thank you for the reviewer’s comments. We have added the reference to the revised manuscript (Line 521).

**Responses to Reviewer #3:**

**#3-1.** *While it is interesting that rG66A resulted in reduced NS1' expression but not NS1 expression, the authors did not discuss nor clarify why the single nucleotide substitution resulted in reduced NS1' expression. This explanation is necessary as all subsequent studies are related to observations found in rG66A.*

**#3-1 Answer:** Thank you for the reviewer's suggestion. NS1' protein is generated by a -1 ribosomal translation frameshift. Bioinformation analyses reveal there are two essential conditions that resulted in the generation of NS1'. One is the conserved slippery hepta-nucleotide (YCCUUUU) acted as canonical frameshift-stimulating motifs, and the other is 3’-adjacent potential pseudoknots near the beginning of the NS2A gene (PMID: 19196463). A66 substitution in NS2A gene located in the first stem of the pseudoknot structure contributed to the GC-rich pseudoknot and resulted in the formation of NS1' (PMID: 26384477; PMID: 22504173). We have added the reference to the revised manuscript (Line 80-82, 245-246).

**#3-2.** *Figure 2E. Why did the authors transfect the last NS1'44aa? While I understand that the 52aa extension of the C-terminus makes NS1' unique from NS1, does the 44aa encode for the active site of NS1' activity? This should be elaborated and discussed.*

**#3-2 Answer:** Thank you for the reviewer’s comments and we are sorry for not expressing ourselves clearly and the mistake. The NS1’ protein is an additional form of NS1 protein with 52aa extension, which includes the N-terminal 9aa of NS2A and 43aa after the frameshift (PMID: 19906906; PMID: 22504173; PMID: 23760245). Therefore, the 43aa are unique to NS1’. The peptide we synthesized includes the unique 43aa along with a cysteine added before to conjugate with KLH, making a total of 44aa. We have changed the description in the revised manuscript. We have expanded our discussion of these concerns mentioned by the reviewer to the Discussion section (Line 470-476, 489-495).

**#3-3.** *Figure 3D, H, and L are missing data points. Does this mean that the experiments are only performed once?*

**#3-3 Answer:** We recognize the reviewer's concerns. Actually, Figure 3D, H and L correspond to Figure 3C, G, and K, respectively, representing the infection ratio of mosquitos in each figure. As a result, each column in Figure 3D, H, and L contains only one point, for example, in Figure 3D, the infection ratio decreases from 81.25% to 51.13%. Meanwhile, the experiments were biologically repeated at least 2 times with similar results.

**#3-4.** *Figure 3C and G. The splitting of the y-axis (especially Figure 3C with 2 split axis) makes the figure hard to interpret, as it is difficult to visualize the distribution of the data. The authors can consider log-transformation if the differences are hard to visualise.*

**#3-4 Answer:** We thank the reviewer for pointing out this issue. We have adjusted the split of the Y-axis to present the data more effectively and visually in Figure 3C and 3G.

**#3-5.** *Figure 5. Besides performing comparisons of the purified NS1' vs BSA under uninfected conditions, the authors should also do purified NS1' vs BSA under infection conditions. This is because the innate immune responses (JAK-STAT, Toll, and IMD) are triggered during virus infection, so this will allow the authors to understand if NS1' affects the induction of these responses which has been shown to impact virus infection outcome.*

**#3-5 Answer:** Thank you for the reviewer’s suggestion. To address this question, we conducted RNA sequencing under infection conditions. In alignment with previous findings, the Toll pathway was downregulated in mosquitoes co-treated with NS1′ and rG66A (Figure 4A-C). This result provides additional evidence that NS1′ indeed promoted viral replication by inhibiting the Toll pathway. We have added the result in the revised manuscript (Line 416-431).

**#3-6.** *Figure 5A. Red and green is very hard to visualise, especially that the gradient of colours span from -3 to 1. Furthermore, the colours are not colour-blind friendly, and do not allow readers to understand the variation of the data. The authors should consider plotting volcano plots, which allows readers to quickly visualise the effect size and significance of the differences.*

**#3-6 Answer:** Thank you for the reviewer’s comments. The RNA sequencing data have been presented by volcano plot in the Figure 4A and 4B. We kept the heatmap to illustrate the downregulation of immune genes but adjusted the color scheme: blue represents downregulation Figure 4C. This modification makes the results more visually accessible.

**#3-7.** *Figure 5B. It is unclear why the dotted lines are drawn at the 0.5 fold-change. Again, plotting* *volcano plots will help clarify the differences better.*

**#3-7 Answer:** Thank you for pointing out the mistake in our manuscript. We have corrected it in the revised Figure 4D and the dotted line is drawn at 1 fold-change. The RNAseq data have been presented by volcano plot in the Figure 4A and 4B.

**#3-8.** *Finally, as the authors have done RNAseq, were there other hits that could be interesting? Could there be other mechanisms involved other than AMPs? If it is only AMPs, would altering the expression of AMPs affect the infection outcome? The authors should report these findings and discuss what they have found.*

**#3-8 Answer:** Thank you for the reviewer’s comments. In the RNA sequencing analysis, the most pronounced expression alterations following NS1' treatment were detected in the Toll pathway and several AMPs, notably DEFA and DEFC (Figure 4A-C). Consequently, our investigation concentrated predominantly on these genes. Furthermore, several genes from the serine protease family were significantly downregulated. Previous studies have shown that these genes are also involved in the regulation of the Toll pathway. To further confirm the role of toll pathway and AMPs in viral infection, we used RNAi technology to silence toll pathway components (*REL1A* and *REL1B*), *DEFC,* and *CECB* in mosquitoes. Three days after silencing the above genes in *Culex quinquefasciatus*, we injected them with a combination of rG66A and NS1'. The results showed that after the genes knockdown, NS1' no longer promoted viral infection (Figure 4E-G). This indicates that NS1' indeed facilitates viral infection by regulating AMPs expression. We have added the result in the revised manuscript (Line 432-440).

**#3-9.** *Authors should also deposit their RNAseq data in GEO or ArrayExpress so others can access the data.*

**#3-9 Answer:** Thank you for the reviewer’s comments. We have deposited the RNAseq data in the GEO database and the accession number is included in the revised manuscript (Line 228-229).
